# Effects of maternal age and environmental enrichment on learning ability and brain size

**DOI:** 10.1093/beheco/arae049

**Published:** 2024-06-17

**Authors:** Náyade Álvarez-Quintero, Sin-Yeon Kim

**Affiliations:** Grupo de Ecoloxía Animal, Centro de Investigación Mariña, Universidade de Vigo, Fonte das Abelleiras, s/n, Vigo, 36310 Pontevedra, Spain; Dipartimento di Biologia, Complesso Interdepartamentale A. Vallisneri, Università di Padova, Via Ugo Bassi, 58b, 35121 Padova PD, Italy; Grupo de Ecoloxía Animal, Centro de Investigación Mariña, Universidade de Vigo, Fonte das Abelleiras, s/n, Vigo, 36310 Pontevedra, Spain

**Keywords:** brain, cognition, enrichment, *Gasterosteus aculeatus*, maternal age, plasticity

## Abstract

It is well known that maternal age at reproduction affects offspring lifespan and some other fitness-related traits, but it remains understudied whether maternal senescence affects how offspring respond to their environments. Early environment often plays a significant role in the development of an animal’s behavioral phenotype. For example, complex environments can promote changes in cognitive ability and brain morphology in young animals. Here, we study whether and how maternal effect senescence influences offspring plasticity in cognition, group behavior, and brain morphology in response to environmental complexity. For this, juvenile 3-spined sticklebacks from young and old mothers (i.e. 1-yr and 2-yr-old) were exposed to different levels of environmental enrichment and complexity (i.e. none, simple, and complex), and their behavior, cognitive ability, and brain size were measured. Exposing fish to enriched conditions improved individual learning ability assessed by a repeated detour-reaching task, increased the size of the whole brain, and decreased aggressive interactions in the shoal. Maternal age did not influence the inhibitory control, learning ability, and group behavioral responses of offspring to the experimental environmental change. However, maternal age affected how some brain regions of offspring changed in response to environmental complexity. In offspring from old mothers, those exposed to the complex environment had larger telencephalons and cerebellums than those who experienced simpler environments. Our results suggest that maternal effect senescence may influence how offspring invest in brain functions related to cognition in response to environmental complexity.

## Teaser

We exposed juvenile sticklebacks of young and old mothers to different experimental environments and found that environmental enrichment improved learning, increased brain size, and reduced aggression, regardless of maternal age. Offspring from old mothers had some brain regions of larger size when exposed to enriched environments, suggesting that maternal age may influence how the brain responds to environmental complexity.

## Introduction

It is now well known that the age of mothers can affect offspring lifespan and other fitness-related traits in many animal species, including humans, leading to the transmission and accumulation of aging effects in subsequent generations (“Lansing effect”: Lansing 1947; Monaghan et al. 2020; Ivimey-Cook et al. 2023). Maternal age-related deterioration directly affects offspring’s condition at birth and life-long performance through the declines in genetic integrity of gametes and non-genetic maternal resources and other substances, such as hormones, antibodies, messenger RNA (mRNA) transcripts, and mitochondrial DNA (mtDNA) copies. Gametes from senescent parents are more likely to carry DNA mutations, impaired mitochondrial function, and shorter telomeres (Gao et al. 2019; Monaghan and Metcalfe 2019; Monaghan et al. 2020). As mothers experience age-related deterioration, their capacity to transfer maternal resources and substances may also decline, showing maternal effect senescence (Moorad and Nussey 2016; Lemaître and Gaillard 2017; Kim et al. 2023; but see Beamonte-Barrientos et al. 2010).

Traditionally, researchers have focused on investigating the negative effects of advanced maternal age at reproduction on offspring physiological condition and life-history traits (reviewed in Monaghan et al. 2020). However, there has been recently a growing interest in the negative effects of marental senescence on the neurodevelopment of offspring and its consequences for cognitive ability and behaviors. For example, studies in small mammals have shown a negative influence of advanced maternal age on offspring cognitive ability (Luo et al. 2017; Merikangas et al. 2017; Sampino et al.2017; Mao et al. 2018), brain structure (Shaw et al. 2012) and synaptic plasticity (Han et al. 2022), which have been associated with de novo mutations and maternally mediated epigenetic changes (Janecka et al. 2017). Similar effects may be possible also in oviparous vertebrates (e.g. fishes, amphibians, and reptiles) because egg quality and composition often change as females age, and these changes negatively affect a variety of offspring traits (Monaghan et al. 2020). Nevertheless, it remains largely unknown to what extent the age of mothers at reproduction can affect offspring’s ability to adjust the development of the brain and behavior in response to environmental conditions.

An organism’s behavioral and neurological phenotype is the result of complex interactions between its genetic inheritance and the environment it experiences during development (West-Eberhard 2003). Although some automated and innate behavioral responses are determined mostly by genes, most behavioral patterns of an individual are modified and fine-tuned through experience (Mery and Burns 2010; Brown 2012). It is now increasingly recognized that in addition to genetic effects, maternal effects also play a crucial role in the phenotypic development of behaviors (Badyaev 2008). Since behavioral responses are controlled by neurological processes in the brain, the development of brains and behaviors occurs in coordination (Shumway 2010; Triki et al. 2022a, 2023). Adaptive behavioral and neural plasticity allows animals to cope with spatial and temporal environmental variation by adjusting their phenotypes to the given conditions (Schlichting and Pigliucci 1998; West-Eberhard 2003). If there is no constraint, animals should exhibit unlimited plasticity, expressing the best trait value in every environment. In general, however, it is difficult for animals to consistently produce the optimum behavior because there are costs of plasticity (DeWitt et al. 1998; Auld et al. 2010). An interesting question is whether individuals vary in the degree of behavioral and neurological plasticity according to maternally inherited condition.

Adaptive behavioral plasticity (and possibly neurological plasticity) can be induced through learning (Brown 2012; Carbia and Brown 2019). Learning may enable individuals to modify or choose their behavioral responses to suit their environments. For example, Trinidadian guppies reduce the costs of anti-predator behavior by learning to distinguish between hungry and satisfied predators and adjust their behavior accordingly (Licht 1989). In captive animals, environmental enrichment may enhance individual learning ability by providing an opportunity to interact with different stimuli and fine-tune the response. Environmental enrichment, defined as an increase in the complexity and novelty of the environment (Hebb 1947; Voss et al. 2013), has been widely shown to bring many benefits to individual health and fitness (Würbel 2001). Environmental enrichment can alter brain structure and function, and such changes are associated with improved cognitive abilities, including learning and memory (Kotrschal and Taborsky 2010; Ebbesson and Braithwaite 2012). Such neurological and behavioral responses to environmental changes can depend on the current condition of the individual (Houston and McNamara 1992; Schlichting and Pigliucci 1998) or even the condition of the mother at reproduction (Mousseau and Fox 1998; Taborsky 2006). Offspring of old mothers may have a limited ability to produce adaptive behavioral plasticity due to the negative effects of maternal effect senescence (Monaghan 2008; Han et al. 2022). It is also possible that they adjust their behavioral and neurological responses to the developmental environment more flexibly than the offspring of young mothers to optimize the investment of relatively limited resources.

Here, we investigate the extent to which maternal age at reproduction influences offspring’s responses to environments with different levels of enrichment. We explore whether maternal age and environment interactively influence the development of cognitive ability, especially inhibitory control and learning, and the brain and whether these changes have consequences for growth and survival. For this, juvenile 3-spined sticklebacks (*Gasterosteus aculeatus*) from a short-living (typically 1-yr in the wild) population were produced from young and old (1-yr and 2-yr-old) mothers by in vitro fertilization (IVF) and then artificially incubated without parental care, which is provided exclusively by males in this species. The 3-spined stickleback has proven to be an excellent model for investigating the plasticity of the traits directly involved in cognitive processes (Bell and Foster 1994; Brydges et al. 2008; Feng et al. 2015). In a parallel experiment, using the same F1 families obtained from the 1-yr-old young females and 2-yr-old senescent females, we demonstrated the presence of maternal effect senescence and showed that the offspring of old mothers had higher rates of malformation and early mortality and had smaller body size at maturity than those of young mothers (Kim et al. 2023). In the present experiment, using the offspring obtained from the parallel study, we experimentally tested whether the interplay between maternal age and environmental enrichment experienced during early life affects inhibitory control and learning rate (in a repeated detour-reaching task), brain morphology, growth, survival, and group behavior (i.e. aggression, exploration, and activity). For this, we performed a factorial experiment using the offspring of young and old mothers, which were exposed to different levels of habitat enrichment (i.e. none, simple, and complex). In the enriched environments, groups of 3 juveniles were maintained in changing habitats over 1 mo. We expected that providing an enriched environment during early development would increase their behavioral and neural plasticity, thus better preparing them to confront the challenges they may encounter in complex and novel environments. We predicted that the degree of plasticity would be limited in the offspring from old mothers, or that this plastic response to environmental challenges would trade off against other developmental functions.

## Material and methods

### Experimental fish

Stickleback populations in Spain, located in the lower latitudes of the species’ European range, exhibit an accelerated life-history trajectory, showing earlier maturation and shorter lifespan, compared to the high-latitude populations (Fernández et al. 2006). In the study population, fish mature from age 9 mo onward and spawn on average six times between February and July. In the natural population, most fish die by the end of the single breeding season (Kim et al. 2015). Thus, the natural lifespan of this population is less than 18 mo in their native habitat. However, under captive conditions in our laboratory, many fish survive and reproduce until the second breeding season because there is no extrinsic mortality by predation, parasitism, extreme weather, and food limitation (Kim et al. 2023).

In the present study, we used F1 individuals obtained by crossing 1-yr-old males and both 1-yr and 2-yr-old (hereafter referred to as young and old) females originally captured from a natural population in Río Sar (Galicia, Spain). The young and old females were in their first and second breeding season, respectively. The breeding design is fully described in a previous study (Kim et al. 2023). Briefly, sticklebacks from the 2019 and 2020 cohorts were captured in March 2020 and February 2021, respectively, and kept under laboratory conditions until the 2021 breeding season. In April 2021, during the peak spawning season, a total of 60 full-sibling families were produced by split-clutch in vitro fertilization (IVF) using 16 young and 15 old females and 22 young males (for IVF protocol, see Kim et al. 2022, 2023). Only one clutch per female was used, and the clutch was divided into 2 sets of 20 to 30 eggs, which were then separately fertilized with sperm from 2 different males. For IVF, we always used fresh sperm extracted from sacrificed males (Kim et al. 2023). The fertilized eggs were left to stand for 20 min and then transferred to a 30-L incubator, where different split clutches were incubated separately. Any confounding effect of parental behavior was removed by IVF and artificial incubation.

Prior to hatching, each F1 family was isolated in a 10-L tank with a sponge filter. Once they hatched and absorbed the yolk sac (i.e. around day 3 after hatching) they were fed daily on a progressive diet of newly hatched *Artemia salina* and a commercial pelleted diet (Gemma Micro, Skretting, Norway). When the juveniles were 2 mo old, they were allocated to 8 L tanks connected to closed water systems, in which water was continuously filtered for nitrification, aerated, and temperature-controlled at 15 °C by water-cooler devices. At age 5 mo, a subsample of 126 individuals from 35 out of the 60 families was selected for the present experiment. Fish from 16 families of old females and 19 families of young females produced by crossing 14 young and 11 old females and 18 young males were used. They were allocated into 10 L independent experimental tanks (34 × 20 × 25 h cm; *N *= 42 tanks) with a sponge filter, each housing 3 individuals of old or young mothers from different families. The lateral walls of the tanks were opaque, preventing visual contact of fish between different tanks. Before transferring to the experimental tanks, the fish were weighed, measured, and permanently marked with color elastomer tags (Northwest Marine Technologies, Shaw Island, WA, United States) under a low dose of benzocaine anesthetic to allow identification. We balanced body size and mass between samples from young and old mothers to control for any confounding effect of maternal age and offspring size (Kim et al. 2023). The survival of fish was monitored daily during the study. Dead fish were stored at −80 °C for molecular sex determination.

### Environmental enrichment

**Fig. 1.**
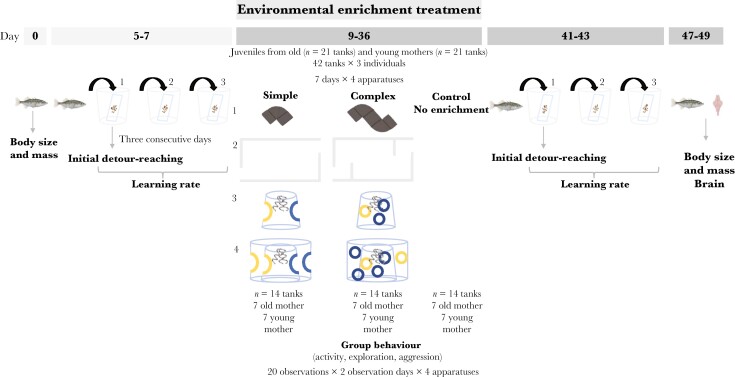
Chronogram of the study. Five days after we measured the fish (i.e. body size), the cognitive ability (i.e. inhibitory control and learning rate) of the experimental fish was individually assessed by a detour-reaching task repeated on 3 consecutive days. Two days later, the experimental tanks containing offspring from old or young mothers were randomly distributed in 3 experimental groups (simple enriched, complex enriched and control no-enriched) and then sequentially exposed to 4 different apparatuses for 4 wk (1 wk per apparatus). In the control group, no apparatus was added. During the exposure of each apparatus (Apparatus 1, from day 9 to 15; Apparatus 2, from day 16 to 22; Apparatus 3, from day 23 to 29; Apparatus 4, from day 30 to 36), the activity, exploration, and aggressive interactions were observed in the experimental tanks. Finally, after the end of the experimental treatment, we evaluated the inhibitory control and learning rate of the experimental fish again in 3 consecutive days (days 41–43). The survival was monitored from the start of the experimental treatment until all fish were euthanized for brain measurements (i.e. from day 9 to 49; 40 d; highlighted in dark gray).

The experimental tanks containing juveniles from old (*N* = 21 tanks), and young (*N* = 21 tanks) mothers were randomly distributed in 3 experimental groups (control, no enrichment; *N* = 14, simple enriched environment; *N* = 14, and complex enriched environment; *N* = 14). As maintaining a certain degree of novelty is important for environmental enrichment (Young 2013), juveniles in the enriched environment groups (hereinafter simple and complex groups) were sequentially exposed to 4 different apparatuses (4 simple and 4 complex) for 1 mo (1 wk per apparatus; 4 treatment weeks), whereas the control tanks were maintained without any apparatus (see Fig. 1 for illustration of the study design). By changing the apparatus every week, we introduced environmental variation and reduced physical and psychological monotony (Young 2013; Näslund and Johnsson 2016), although this treatment may also lead to frequent perturbations (Lee and Berejikian 2008). The apparatuses used for the simple and complex groups had similar designs but different levels of complexity (see Fig. 1 and Supplementary Fig. S1). Our study population comes from a structurally complex river and shows mainly benthic behavior. The apparatuses were designed to combine artificial structures with sensory stimuli as explained below with more details. Thus, the experimental sticklebacks were exposed to 4 different apparatuses to increase the habitat complexity and stimulate the visual, somatosensory, and vestibular sensory systems, each of which are involved in vision, somatic senses, and spatial orientation (Cancedda et al. 2004; Wells 2009). Our treatment with apparatuses may provide shelter for fish and reduce social stress and aggression between individuals (Slavík et al. 2012; Santos et al. 2013). It may also reduce swimming rate, metabolic rate, and physical deterioration (Fischer 2000; Berejikian and Tezak 2005; von Krogh et al. 2010). Increasing complexity into the tanks perhaps allows more fish to find shelter (Wocher et al. 2011).

Apparatus 1 consisted of a dark gray PVC pipe with a curvature of 87°, a diameter of 4 cm, and a total length of 6.5 cm for the simple group, and a longer and more complex tunnel made of 2 connected pipes for the complex group, both of which had 2 entrances for fish (Fig. 1a and b; see also Supplementary Fig. S1a and b). In both apparatuses, once a fish entered the tunnel, it could not see the opposite exit and could remain out of sight of the group.

Apparatus 2 used for the simple group consisted of a transparent plastic cup 13 cm high with a diameter of 6.5 cm at the bottom and 9 cm at the top placed upside down. The cup had 2 circular entrances (diameter 6 cm), one of them outlined in blue and the other in yellow (Fig. 1c, see also Supplementary Fig. S1c). For the complex group, we used the same cup but with 3 smaller entrances of a diameter of 3 cm, two of which were true entrances outlined in blue, and the other was a closed false entrance outlined in yellow (Fig. 1d and Supplementary Fig. S1d). Coils made of aluminum foil were placed on the top of the cup as an attractant and shelter in both experimental groups (Merilä 2012) (Fig. 1c and d; see also Supplementary Fig. S1c and d).

Apparatus 3 consisted of 2 L-shaped opaque plastic panels of 20.5 cm length, 15 cm width, and 20 cm height that reached the water’s surface. For the simple group, the 2 panels were placed together to form a box-shaped simple maze with 2 entrances (Fig. 1e and Supplementary Fig. S1e). For the complex group, we used the same L-shaped panels but with a third barrier in the center to create a more complex maze (Fig. 1f and Supplementary Fig. S1f).

Apparatus 4 was a 2-layer cylinder, constructed by surrounding Apparatus 2 with a transparent cylinder made of an acetate sheet, with the same entrances as those of Apparatus 2 of each experimental group. For the simple group, the entrances of each layer (i.e. acetate sheet and cup entrances) were overlapped while for the complex group were placed in a different position (see Fig. 1g and h; Supplementary Fig. S1g and h and Fig. S2).

### Detour-reaching task

**Fig. 2.**
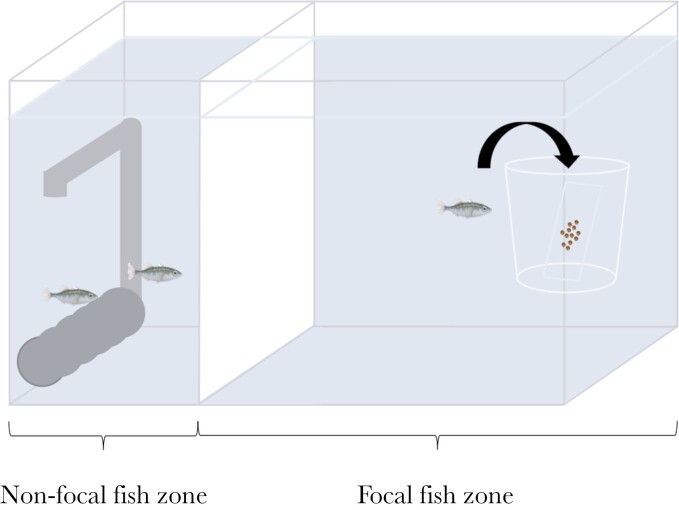
Illustration of an experimental tank during the detour-reaching task. The divider to separate the focal and non-focal fish was positioned, leaving two-thirds of the tank as the focal zone where the transparent plastic cup used as the barrier was placed. To access the food reward, the focal fish must swim into the cup through the open top.

The inhibitory control (i.e. initial detour-reaching score) and learning rate of the fish was individually assessed before (*N* = 126) and after (*N* = 105) the experimental treatment (when fish were 156 ± 6.35 and 192 ± 6.27 d old, respectively) by a detour-reaching task in which the fish needed to inhibit an ineffective prepotent behavior when attempting to achieve a goal (Boogert et al. 2011; MacLean et al. 2014). We used a transparent plastic cup (5 cm diameter base, 7 cm tall) with a 6-cm diameter opening as a barrier (see Fig. 2). During each test, the focal fish had to find the entrance to access the food reward inside the cup, which was visible from all directions through the transparent cup (Minter et al. 2017; Álvarez-Quintero et al. 2021). The detour-reaching task was performed repeatedly on 3 consecutive days (i.e. first, second, and third test day) both before and after the experimental treatment (see Fig. 1). The repeated tests allowed us to assess the learning rate in each individual. Most species are capable of learning to inhibit their grabbing response and instead detour the opening of the enclosure to reach the object (Amici et al. 2008; Vlamings et al. 2009).

Before the first detour-reaching task test, the fish were exposed to the cup (without food reward), which was placed in their respective experimental tanks for 24 h to avoid neophobia (Álvarez-Quintero et al. 2020). We did not feed fish during this 24-h period to increase their motivation for the food reward. After the 24-h exposure, we conducted the first test in which the fish tried to access the cup once the food was provided. The same food pellets as the habitual diet were attached to a microscope slide (24 × 60 mm) using petroleum jelly and this slide was placed inside the cup. Because there was a group of 3 fish in each tank while testing a focal fish, the other 2 fish were isolated by using an opaque divider to prevent them from watching the focal fish’s performance (see Fig. 2). Before the first test, we introduced the divider in the tank for 3 h a day during 3 consecutive days to habituate the fish to its presence. The focal fish was identified by the color elastomer tag and observed for up to 120 min to record the time taken to enter the cup. In cases where fish did not successfully enter the apparatus, we assigned the maximum time (120 min) before/after the experimental treatment (first test: 29 out of 126 and 10 out of 105 fish; second test: 19 out of 126 and 7 out of 105 fish; third test: 11 out of 125 and 9 out of 105 fish). Once the test ended, the fish was moved to the non-focal zone, and the used microscope slide was removed. The same test was performed individually for the other 2 fish. The test order of 3 individuals was changed in the following days. Thus, each fish was tested in all 3 orders during 3 consecutive tests. The tests were always conducted between 9 am and 4 pm. After each test day, we removed the divider and the cup, and the fish were fed normally.

### Activity, exploration, and aggressive interactions

Behaviors of the fish were observed 2 to 3 min after we introduced each apparatus (hereinafter first sampling day) and after 3 d of exposure to the apparatus (hereinafter second sampling day) in the simple and complex groups. Fish in the control group were also observed on the same days. The levels of activity, exploration, and aggression of fish in each tank were registered by 3 trained observers using instantaneous scan sampling for 20 min at 1-min intervals (i.e. 20 observations per tank). Each behavior was always registered by the same observer. Observers were blinded to maternal age, but not to environmental enrichment during behavioral observations due to the presence of the apparatus in the tanks. Before the first sampling day, we introduced a gloved hand to the control tanks in the same way as to the simple and complex enrichment groups, simulating apparatus installation, to control for its effect (if any). Although some individuals were identified by their elastomer tags during the scan sampling, we did not record fish identity because the detection of individual fish would be biased due to differences in the visibility of different color tags. Behaviors were always registered between 10 and 12 a.m. The observers were always placed on the same side of the tanks and 2 m away so as not to interfere with the normal behavior of the fish.

To evaluate whether the experimental treatment affected fish activity, we registered the number of fish swimming in each tank during the scan sampling. An individual was considered swimming if it was changing position at the time of observation, either propelling itself forward or backward or moving up and down. To examine the level of exploration, we registered the number of fish actively exploring (i.e. touching, entering, approaching, or observing the apparatus within their body length distance). Exploratory behavior was not assessed in the control tanks without apparatus. To quantify aggression, we registered whether any fish in the tank showed aggressive behaviors, such as biting, chasing, and lunging toward others. Lunging is distinguished from non-aggressive approaching because it is frequently accompanied by an attempt at biting and alters the other’s behavior. For statistical analyses, we used the maximum number of fish swimming or exploring out of the 20 observations for each sampling day divided by the total number of fish in the tank (range 2 to 3), and the proportion of observations (out of a total of 20 for each sampling day) in which at least one fish showed aggressive behaviors (for swimming and aggression: 4 apparatuses × 2 sampling days × 42 tanks, *N* = 336; for exploration: 4 apparatuses × 2 sampling days × 28 tanks, no observation for the control tanks, *N* = 224). The maximum number was correlated with the average number of fish swimming or exploring during the observations (both *R* > 0.373 and *P* < 0.001).

### Brain measurements

Three days after the last detour-reaching task test (average fish age: 195 ± 6.27 d), all fish that survived (*N* = 101) were euthanized by an overdose of benzocaine anesthetic. Each fish was weighed to the nearest 0.01 g with a digital balance, and its standard length (hereinafter body size) was measured to the nearest 0.5 mm with a caliper. Then, the brain was dissected from the cranium, and a drop of phosphate-buffered saline solution was added to prevent the brain from drying out during the procedure, which could affect its volume. Digital photographs of the brain were taken from 3 viewpoints (dorsal, lateral, and ventral; see Supplementary Fig. S3) by using a digital stereomicroscope (Leica S9i). All lateral images were taken of the right hemisphere (Pollen et al. 2007). Skeletal muscle samples were also collected and stored at −80 °C for molecular sex determination.

The size of the whole brain and 4 different brain regions—telencephalon, optic tectum, cerebellum, and hypothalamus—were measured from digital photographs using ImageJ software (Rasband 1997). The width (W), height (H), and length (L) of each region were taken and defined following the measurement procedure detailed in Pollen et al. 2007. The volume (V) of each brain region was calculated as V = (L × W × H) π/6. For paired structures, we used a doubled volume estimate of the same lateral side measurements (Pollen et al. 2007). The whole brain volume was estimated as the sum of all different brain region volumes (Pollen et al. 2007). Because some brain regions were damaged during dissection or were not properly positioned on the digital image (telencephalon: *N* = 2; optic tectum: *N* = 3; cerebellum: *N* = 11 and hypothalamus: *N* = 11 out of 101 individuals), the whole brain volume was estimated only in the samples with all brain region volumes estimated (*N* = 86). The whole brain volume and different brain region volumes were positively correlated to each other (all *P* < 0.001; see Supplementary Fig. S4), except for the correlation between the hypothalamus and the optic tectum (*R* = 0.069, *P* = 0.519).

### Sex determination

All the experimental fish (*N* = 126), including those that died during the course of the experiment, were sexed following the molecular methodology and primer sequences described by Velando et al. 2017. The method is based on polymerase chain reaction (PCR) to amplify part of the 3’UTR of the NADP-dependent isocitrate dehydrogenase locus. The DNA products were then run on a 2% agarose gel and stained with Greensafe Premium (NZYtech, Portugal).

### Statistical analyses

All statistical analyses were performed in R v.4.2.2. Linear mixed-effect models (LMMs) and generalized linear mixed models (GLMMs) were performed using the *lmer* and *glmer* functions of the *lme4* package (Bates et al. 2015). All random effects in the LMMs or GLMMs were random intercept unless specified. In LMMs, all data have equal variance between treatments (Levene’s Test) and the residuals from the linear regression models were normal (Shapiro–Wilk test). In the Cox proportional hazard model (CPHMs), the proportional hazards, influential observations, and non-linearity assumptions were tested by residual methods using *ggcoxzph*, *ggcoxdiagnostics*, and *ggcoxfunctional* functions of the *survminer* package. All GLMMs were checked for overdispersion using the *overdis_fun* function. The significance of the term was determined by the *F* test with Satterthwaite approximation for degrees of freedom (using the *anova* function of the *lmerTest* package; Type III sums of squares) in LMMs (Kuznetsova et al. 2016) and by the Likelihood Ratio Test (LRT) in GLMMs and CPHM. In all models detailed below, we also tested the interaction between maternal age (young and old) and environmental enrichment treatment (control, simple and complex enrichment). If the interacting effect was statistically not significant, we also tested the effects of the main terms in an additional model, excluding the interaction because their inclusion may produce inaccurate estimates for the main effects (see Engqvist 2005). Post-hoc comparisons were performed using Tukey’s post-hoc test using the *TukeyHSD* function. The standardized coefficients (β) and their confidence intervals (95% CIs) were calculated as a measure of effect sizes in all models by using the *effectsize* package (Ben-Shachar et al. 2020). The *P*-values were adjusted using the false discovery rate (FDR) method for multiple comparisons with the *p.adjust* function (i.e. analyses of activity, exploration, aggression and whole brain, optic tectum, hypothalamus, telencephalon, and cerebellum volumes). For easy interpretation, all figures present the original data without transformation.

We compared the initial body size and mass of fish among the experimental groups by using LMMs including maternal age, environmental enrichment treatment, and their interaction as fixed effects and mother and father identities as random effects [initial body size or body mass = maternal age + treatment + maternal age × treatment + (mother id) + (father id)].

The inhibitory control (i.e. initial detour-reaching score; first score of 3 consecutive detour-reaching task tests), before and after the experimental treatment were analyzed in separate LMMs, including maternal age, environmental enrichment treatment, their interaction, and sex, as fixed terms, and mother, father, and tank identity as random effects [initial detour-reaching score = maternal age + treatment + sex + maternal age × treatment + (mother id) + (father id) + (tank id)]. Prior to statistical analyses, we log-transformed the detour-reaching task scores before and after the experimental treatment to improve data distribution and meet model assumptions of normality [log (0.25+χ)]. Individual learning rate (i.e. the ability to learn a task over time) before and after the experimental treatment were analyzed in separate LMMs, including maternal age, environmental enrichment treatment, test day (first, second, and third), their interactions, and sex, as fixed terms, and the individual identity (intercept) and the individual change across tests (slope) as random effects (i.e. random regression). Mother, father, and tank identities were also included as random effects [detour-reaching task score = maternal age + treatment + test day + sex + maternal age × treatment × test day + maternal age × treatment + maternal age × test day + treatment × test day + (1 + test day׀individual id) + (mother id) + (father id) + (tank id)].

We tested whether group behaviors (i.e. activity, exploration, and aggression) differed between the experimental groups and maternal ages, and whether they changed across sampling days (first and second sampling day in each apparatus) and treatment weeks (1 to 4 wk) by using GLMMs. The maximum number of fish swimming and exploring the apparatus in the tank were analyzed in GLMMs with a binomial error distribution and logit link function while the proportion of observations in which at least one fish was involved in aggressive interactions was analyzed in a GLMM with beta distribution (Crawley 2012; Harrison 2015) using the *glmmTMB* package (Brooks et al. 2017). Maternal age, environmental enrichment treatment, and their interaction, sampling day, and treatment week were included as fixed effects. Tank identity was included as a random effect [group behavior = maternal age + treatment + sampling day + treatment week + maternal age × treatment + (tank id)].

Fish growth was analyzed using repeated measures of body size and mass in LMMs. In these analyses, maternal age, environmental enrichment treatment, time of measurement (before and after the treatment), their interactions, and sex were included as fixed effects. Mother and father identities were included as random effects, and individual and tank identity as nested random effects [size or mass = maternal age + treatment + time + sex + maternal age × treatment × time + maternal age × treatment + maternal age × time + treatment × time + (mother id) + (father id) + (tank id) + (1|tank id/individual id)]. Survival trajectories of the experimental fish from the beginning of the experimental treatment to the end of the study (i.e. euthanasia for brain measurements) were analyzed using a CPHM, including maternal age, environmental enrichment treatment and their interaction, and sex as fixed effects, and mother, father and tank identity as random effects [survival = maternal age + treatment + sex + maternal age × treatment + (mother id) + (father id) + (tank id)].

The whole brain and brain region volumes were analyzed in LMMs, including maternal age, environmental enrichment, sex, and body size after the treatment as fixed effects, and mother, father, and tank identities as random effects [whole brain or brain region volume = maternal age + treatment + sex + body size + maternal age × treatment + (mother id) + (father id) + (tank id)]. We also analyzed how the individual initial detour-reaching score and learning rate after treatment were related to the size of the whole brain and 4 different brain region volumes—telencephalon, optic tectum, cerebellum, and hypothalamus—in separate linear models (LMs), each including initial detour-reaching score or learning rate as independent variable

## Results

### Initial differences in body size or mass

There was no difference in initial body size (LMM; maternal age × treatment: *F*_2, 109.51_ = 2.86, *P *= 0.062) or body mass (LMM; maternal age × treatment: *F*_2, 108.73_ = 2.78, *P* = 0.066) between the experimental groups prior to the experimental treatment. The main terms, maternal age, and environmental enrichment treatment were also statistically not significant (all *P* > 0.285).

### Initial detour-reaching and learning rate

**Fig. 3.**
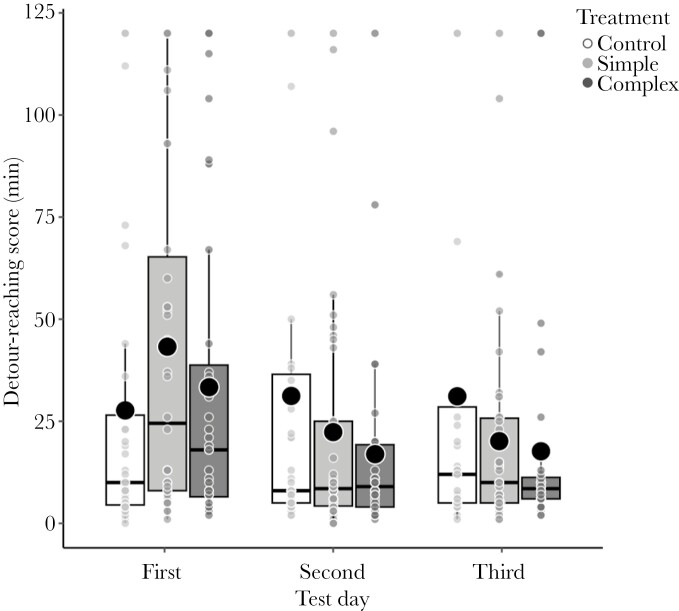
Detour-reaching task scores (time to enter the cup in minutes) over repeated test days (first, second, and third) after the environmental enrichment treatment (control, *N* = 35; simple, *N* = 38; complex, *N* = 32). Small dots represent individual values. Horizontal lines and dots in boxplots are medians and means, and the extend of boxes and whiskers indicate the 25–75th percentiles of the data and 1.5 inter-quartile ranges, respectively.

There was no difference in the initial detour-reaching score (LMM; maternal age × treatment: *F*_2, 111.23_ = 0.91, *P* = 0.405) and learning rate (LMM; maternal age × treatment × test day: *F*_4, 237.45_ = 0.78, *P* = 0.536) between the experimental groups prior to the experimental treatment. Fish from all experimental groups improved their performance on the detour-reaching task throughout repeated tests before the treatment (*P* < 0.001; Supplementary Table S1). Neither the maternal age, environmental enrichment treatment, sex nor the 2-way interactions between maternal age, treatment, and test day (in the learning rate model) affected the initial detour-reaching and learning rate before the treatment (all *P *> 0.084; Supplementary Table S1). However, after 1 mo of environmental enrichment treatment, there was a significant difference in learning rate between the environmental enrichment groups (*P* = 0.048; Supplementary Table S1), while initial detour-reaching (i.e. the first score) was not affected by the treatment (*P* = 0.094; Supplementary Table S1). The fish from the simple and complex groups improved the detour-reaching score from the first to the third test (on average 23.13 and 15.69 min less, respectively), while the control fish did not (on average 3.45 min longer) (Fig. 3). There was no effect of maternal age, environmental enrichment treatment, sex, and maternal age × treatment on the initial detour reaching after the treatment (all *P* > 0.094). Neither maternal age, its interactions with treatment, test day nor sex influenced the learning rate after the treatment (all *P* > 0.239; Supplementary Table S1).

### Group activity, exploration, and aggressive interactions

**Fig. 4.**
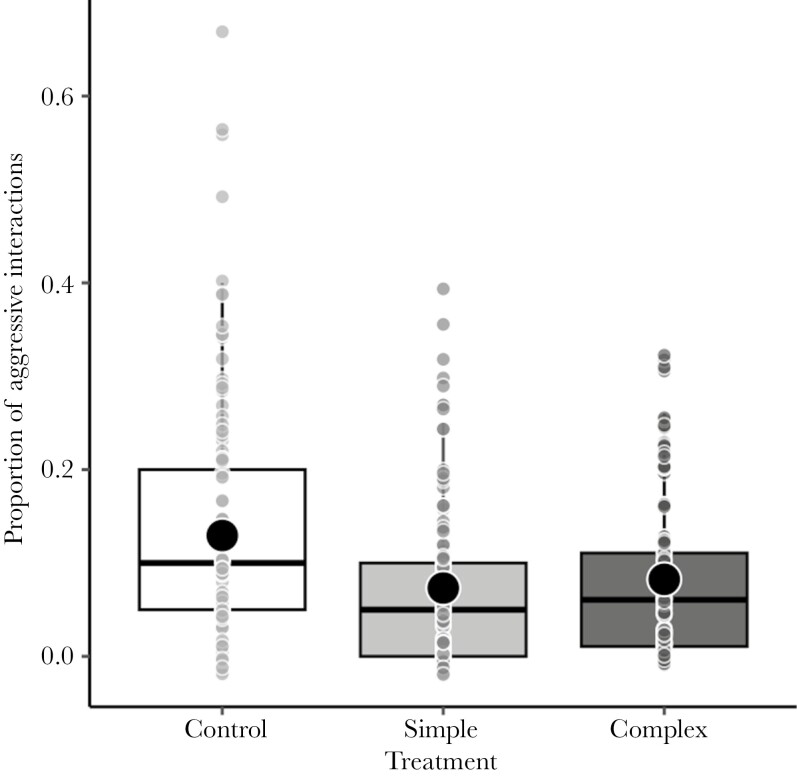
Proportion of observations in which at least one aggressive interaction between individuals was observed in the control, simple, and complex groups. Small dots represent individual values. Horizontal lines and dots in boxplots are medians and means, and the extend of boxes and whiskers indicate the 25–75th percentiles of the data and 1.5 inter-quartile ranges, respectively.

There were significant effects of sampling day on activity, exploration, and aggressive interactions (*P *= 0.018; *P *= 0.027 and *P *< 0.001, respectively; Supplementary Table S2). On the first day of sampling (i.e. the first day of exposure to the apparatuses), more fish swam and explored and more aggressive interactions were observed compared to the second sampling day (i.e. third day of exposure to the apparatuses). However, after correcting for multiple comparisons only sampling day on exploration resulted significant (*P* = 0.018; FDR-adjusted *P* = 0.054, *P* < 0.001; FDR-adjusted *P* < 0.001 and *P* = 0.027; FDR-adjusted *P* = 0.063, respectively). There was no effect of maternal age, environmental enrichment treatment, and their interaction on activity and exploration (all *P* > 0.106), but the control fish showed a higher level of aggression (i.e. the proportion of observation in which aggressive interactions were observed) than the fish in the enriched groups (*P* < 0.001; FDR-adjusted *P* = 0.003; Supplementary Table S2; Fig. 4). Neither the maternal age nor their interaction with treatment influenced aggressive interactions (all *P* > 0.102). The levels of activity, exploration, and aggression differed among the treatment weeks, but a significant difference was maintained only in aggression after p-adjustment (*P* = 0.035; FDR-adjusted *P* = 0.063, *P* = 0.037; FDR-adjusted *P* = 0.074 and *P* < 0.001; FDR-adjusted *P* < 0.001, respectively). The levels of aggression significantly increased across the treatment week. The proportion of observations of aggressive behavior, fish swimming, or exploring was not related to the survival (i.e. the proportion of dead fish per tank).

### Growth and survival

**Fig. 5.**
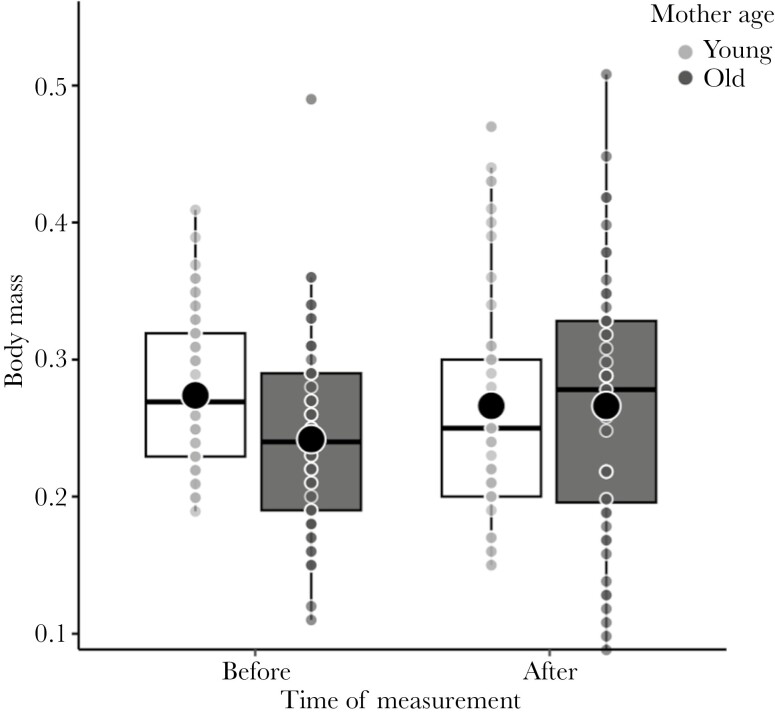
Body mass measured before the experiment (*N* = 126) and at the end of the study when fish were sampled for brain analyses (*N* = 101) in fish from the young and old mothers. Small dots represent individual values. Horizontal lines and dots in boxplots are medians and means, and the extend of boxes and whiskers indicate the 25–75th percentiles of the data and 1.5 inter-quartile ranges, respectively.

**Fig. 6.**
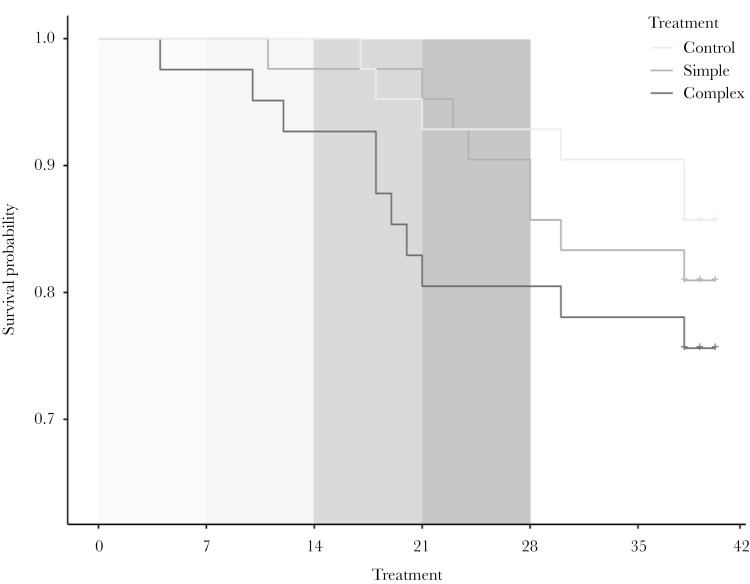
Estimated survival functions of sticklebacks reared in the control (*N* = 42), simple (*N* = 42) and complex (*N* = 42) groups during the study. The gray shadows indicate the period of the experimental treatment in which fish were exposed to apparatuses 1, 2, 3, and 4 (from light to dark gray, respectively) in the simple and complex environmental enrichment groups. The control fish were not exposed to any apparatus. Crosses mark censorship events, i.e. individuals still alive at the end of the monitoring period and sampled for brain analyses.

Growth trajectories differed between fish from young and old mothers in body mass (time × maternal age: *P* = 0.035; Supplementary Table S3) but not in size (time × maternal age: *F*_1, 105.89_ = 2.168, *P* = 0.144). Fish from old mothers gained more weight than those from young mothers during the experiment (Fig. 5). Neither the environmental enrichment treatment nor their interactions with time and maternal age influenced growth rate in body mass or size (all *P* > 0.079). Sex did not affect growth trajectories in body mass and size (both *P* > 0.158). Analysis of survival from the onset to the end of the study (when fish were euthanized for brain analyses; 39.14 ± 0.81 d) was not affected by environmental enrichment treatment, maternal age, and their interaction (CPHM; treatment × maternal age: LRT, χ²₁= 0.86, *P *= 0.650; treatment: *P *= 0.424, maternal age: *P *= 0.808; *N* = 125 individuals; Supplementary Table S4; Fig. 6). However, survival curves show that the fish survival rate to the end of the exposure to Apparatus 3 (i.e. day 21) was apparently lower in the complex group compared to the simple and control groups, and this difference was statistically significant (*P *= 0.040; Supplementary Table S4). This difference was mainly due to a high mortality rate in the complex group during the exposure to Apparatus 3. The survival rates were not influenced by sex (both *P* > 0.261).

### Brain size

**Fig. 7.**
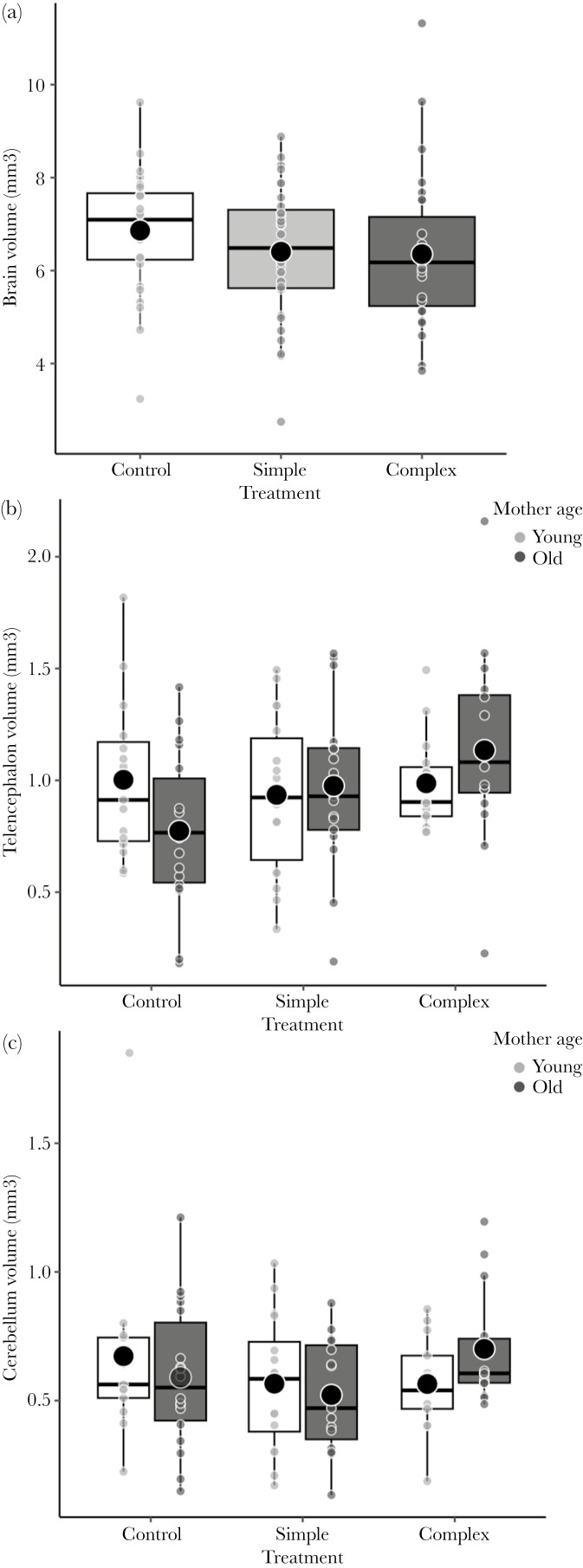
(a) Whole brain volume of fish in the control, simple, and complex groups. (b) Telencephalon volume and (c) cerebellum volume of fish from young and old mothers reared in the control, simple, and complex groups. Small dots represent individual values. Horizontal lines and dots in boxplots are medians and means, and the extend of boxes and whiskers indicate the 25–75th percentiles of the data and 1.5 inter-quartile ranges, respectively.

The analysis of the whole brain size showed that fish in the complex group had bigger brains than those in the simple and control groups (*P* = 0.037; FDR-adjusted *P* = 0.102; Supplementary Table S5; Fig. 7a), although the effect was not significant after correction for multiple comparisons. The treatment had a maternal age-specific effect on the volume of the telencephalon and cerebellum (telencephalon: *P* = 0.010; FDR-adjusted *P* = 0.037; cerebellum: *P* = 0.019; FDR-adjusted *P* = 0.059; Supplementary Table S5). In fish from old mothers, the complex group had bigger telencephalons than the control no-enriched group (Tukey post-hoc test; *P* = 0.032; Fig. 7b), but there was no difference between the complex and simple groups (Tukey post-hoc test; *P* = 0.863). In fish from old mothers, the complex group also had the largest cerebellums (Fig. 7c), although the post-hoc comparisons were not significant (Tukey post-hoc tests: complex vs. control; *P* = 0.180 and complex vs. simple; *P* = 0.224). The optic tectum or the hypothalamus were not affected by environmental enrichment, maternal age, and their interactions (all *P* > 0.076). There was no sex difference in all analyses (all *P* > 0.107; Supplementary Table S5). The volumes of the whole brain and different brain regions were positively correlated with the body size measured after the experimental treatment (all *P *< 0.001).

### Initial detour-reaching, learning rate, and brain

Fish with better initial detour-reaching after the experimental treatment showed larger brains and brain regions except for optic tectum (LMs; whole brain: *F*_1, 84_ = 7.52, *P* = 0.007; FDR-adjusted *P* = 0.035; telencephalon: *F*_1, 97 =_ 10.18, *P* = 0.002; FDR-adjusted *P* = 0.020; cerebellum: *F*_1, 88_ = 5.96, *P* = 0.017; FDR-adjusted *P* = 0.056; and hypothalamus: *F*_1, 88_ = 5.33, *P* = 0.023; FDR-adjusted *P* = 0.057; optic tectum: *F*_1, 96_ = 1.88, *P* = 0.174; FDR-adjusted *P* = 0.248). However, learning rate was not related to brain size or any of the brain regions measured (LMs; whole brain: *F*_1, 84_ = 0.97, *P* = 0.326; FDR-adjusted *P* = 0.362; telencephalon: *F*_1, 97_ = 3.10, *P* = 0.081; FDR-adjusted *P* = 0.135; cerebellum: *F*_1, 88_ = 3.62, *P* = 0.060; FDR-adjusted *P* = 0.120; hypothalamus: *F*_1, 88_ = 1.04, *P* = 0.311; FDR-adjusted *P* = 0.362; optic tectum: *F*_1, 96_ = 0.70, *P* = 0.404; FDR-adjusted *P* = 0.404).

## Discussion

Our results show no evidence for interacting effects of maternal age and environmental enrichment on inhibitory control, learning rate, and group behaviors in juvenile 3-spined sticklebacks. However, after 1 mo of experimental treatment, fish living in enriched environments showed greater learning rates and had larger brains. Environmental enrichment also led to a reduction in aggressive behavior at the group level. Although there was no evidence that maternal age influences the ability to respond to environmental changes through behavioral plasticity, maternal age did influence how some regions of the brain change in response to environmental complexity. Overall, our results highlight the effects of environmental enrichment on behavior, cognition, and brain size in captive fish and suggest that offspring of old mothers invest in brain regions according to environmental complexity.

Our results showed no direct negative effect of maternal age at reproduction on inhibitory control and learning rate of juvenile sticklebacks. Offspring from young and old mothers showed similar initial detour-reaching scores and similarly improved their performance over repeated tests when exposed to enriched environments. This lack of effects could have arisen via selective disappearance if, for instance, juveniles from older mothers, which had lower survival prospects in our population (Kim et al. 2023), also had lower cognitive ability. It is also possible that maternal age affects other cognitive traits and functions than those captured by the detour-reaching task used in this study (Rowe and Healy 2014). Further studies using different cognitive assays are needed to confirm the effect of maternal age on offspring cognitive function.

In accordance with previous studies of other teleost species (Strand et al. 2010; Salvanes et al. 2013; Carbia and Brown 2019; but see Martinez et al. 2016), sticklebacks exposed to environmental enrichment, either simple or complex, showed greater learning rates compared to those in the control non-enriched tanks. Interestingly, sticklebacks in the enriched environments improved only learning ability but not inhibitory control, as also shown in a previous study of guppies (Montalbano et al. 2022). Our environmental enrichment treatment consisted of providing fish with a novel and changing environment, and this probably has improved their adaptability to novel situations, thereby enhancing their learning ability. It is also possible that the cognitive mechanisms recruited to solve a problem in the first encounter and through learning differ (Kabadayi et al. 2018). It is perhaps unlikely that experience with the detour-reaching task prior to the treatment allowed the experimental fish to solve it more easily, otherwise, their initial inhibitory control performance would be comparable to that of the control fish.

In addition to improving cognitive performance (i.e. the ability to improve in an inhibitory control task over time), the environmental enrichment treatment also influenced behaviors in different contexts. Sticklebacks in the enriched groups, both simple and complex, showed lower levels of aggression, but the levels of activity and exploration did not differ between the treatment groups. The environmental enrichment probably reduced aggressive interactions by restricting visual contact between individuals and providing shelter. Although similar habitat enrichment has generally been shown to reduce aggressive interactions (Barley and Coleman 2010; Torrezani et al. 2013), some studies have reported increased aggression in enriched environments, especially in species with territoriality or dominance hierarchy (Woodward et al. 2019). Three-spined sticklebacks, especially males, turn territorial and aggressive when sexually mature, but juveniles show only mild aggressive interactions in shoals and no territorial behavior. Thus, for the experimental fish in our study, the apparatus used for environmental enrichment were objects to hide or explore but not to defend (our observation).

Our survival analyses showed that neither the environmental enrichment nor the maternal age affected the survival rate of the experimental individuals throughout the whole period of the experiment. However, the individuals exposed to a complex enriched environment showed a higher early mortality rate (i.e. until day 21). This is in accordance with our previous study of juvenile sticklebacks, showing that cognitive challenges can result in increased mortality (Álvarez-Quintero et al. 2020). Although there was no maternal age effect on survival, juveniles from old mothers gained more weight during the experiment. Since growth was measured only in individuals surviving to the end of the study, and early mortality differed across the environments, it is possible that selective mortality of slow-growing offspring of old mothers induced this result. Although we have no further data to support this possibility, our parallel study showed that old mothers produced less viable offspring with slow growth and increased early mortality (Kim et al. 2023). It is also possible that the weight gain in the offspring of older mothers during the experiment was due to rapid compensatory growth (Kim et al. 2019). Our data, showing no relationship between group behaviors (aggression, activity, and exploration) and survival, suggest that treatment effects are probably not due to differential mortality mediated by shoal behavior or social stress.

In fishes, the brain continuously grows during their lifetime (Zupanc 2001). Brains are highly plastic and respond adaptively to the environment. For example, previous studies of sticklebacks and other fish species have reported positive effects of enrichment on brain development (Kihslinger and Nevitt 2006; Näslund et al. 2012; Herczeg et al. 2015). Similarly, our results showed that individuals from the complex enriched group had larger relative brain sizes. Our results revealed maternal-age-specific effects of environmental enrichment on telencephalon and cerebellum volumes. Offspring from old mothers exposed to complex environments had bigger telencephalons and cerebellums than those from old mothers reared in simple or control environments. The fish telencephalon is considered the main center for cognition and is involved in spatial learning, memory, and inhibitory control (von Krogh et al. 2010; Salvanes et al. 2013; Triki et al. 2022b), while the cerebellum influences cognition by controlling mainly motor coordination and navigation abilities (Kotrschal et al. 1998; Rodríguez et al. 2005; Ebbesson and Braithwaite 2012). Given that increased investment in brain structures and related functions is costly and may divert resources away from other vital functions (Mery and Kawecki 2003; Fisher et al. 2006), the plasticity in brain size and cognition in response to environmental challenges should increase individual fitness (Burger et al. 2008). This is especially true for those born in poor condition or with less resources provisioned by the mother. Perhaps for this reason, offspring from old mothers show brain size plasticity, increasing investment in brain regions related to cognitive ability only when exposed to complex environments, whereas offspring from young mothers express similar brain phenotypes across different environments.

In conclusion, this study indicates that environmental enrichment not only reduces aggressive interactions in fish shoals but also stimulates individual learning ability and brain development. Our fast- and short-living study population allowed us to experimentally test the consequences of maternal senescence for offspring cognition and brain development. Contrary to the widely held belief that advanced maternal age impairs cognitive functions, our findings suggest no overall negative effects of maternal senescence on offspring cognition. Although this population has evolved to live fast and die before age 2 yr, and offspring of old females outliving the expected lifespan indeed suffered reduced early viability (Kim et al. 2023), maternal age at reproduction perhaps has a limited effect on offspring performances beyond this vulnerable early developmental stage. Interestingly, however, our results suggest that maternal effect senescence may influence how offspring invest in brain functions in response to environmental changes. Future studies should measure the costs and benefits of plasticity in brain and cognitive functions in changing environments and explore how maternal age and phenotype influence offspring plasticity to improve our understanding of maternal effect senescence in offspring cognition.

## Supplementary Material

arae049_suppl_Supplementary_Figures_S1-S4_Tables_S1-S5

## Data Availability

Analyses reported in this article can be reproduced using the data provided by Álvarez-Quintero and Kim (2024).
