# Supplementary material for: Effects of maternal age and environmental enrichment on learning ability and brain size
Source: Behav Ecol. 2024 Jun 17;35(4):arae049. doi: 10.1093/beheco/arae049 (PMC11215699; doi:10.1093/beheco/arae049)
Supplement: arae049_suppl_Supplementary_Figures_S1-S4_Tables_S1-S5 [file arae049_suppl_supplementary_figures_s1-s4_tables_s1-s5.docx]

**Effects of maternal age and environmental enrichment on learning ability and brain size**

**SUPPLEMENTARY INFORMATION**

**Figure S1.** Photographs of the experimental set-up of the fish tanks with the apparatuses 1, 2, 3, and 4 designed for the simple (a, c, e, and g) and complex (b, d, f, and h) enriched groups. In the control (i.e. not enriched) group, no experimental apparatus was added to the tanks. The order of the apparatus in this figure corresponds to the chronological order used in the experiment. The photographs also show the sponge filter located inside the tank, which was present in the control group, too.


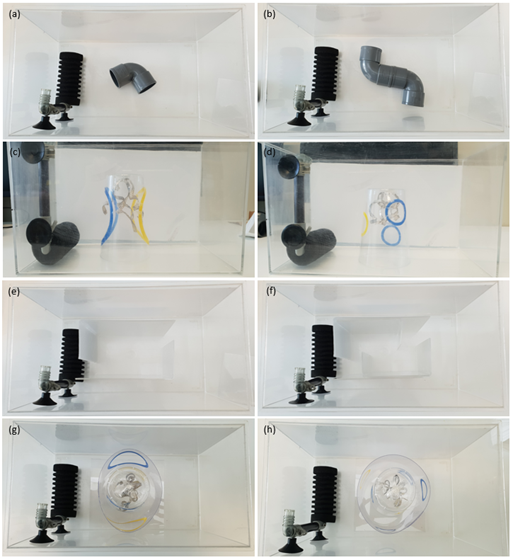


**Figure S2.** Photograph of one of the complex enrichment treatment tanks with apparatus 4 inside. The red circles surround the fish inside the apparatus.


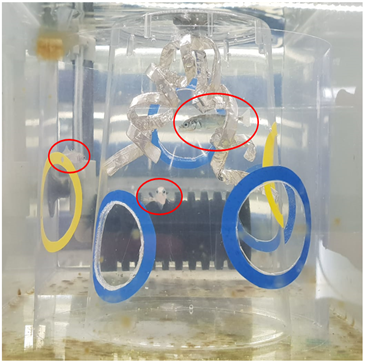


**Figure S3.** Illustration of the measurements taken from (a) dorsal, (b) ventral and (c,d) lateral image to determine the size of various brain structures from *G. aculeatus*. W, H and L refer to width, height and length, respectively. 1: telencephalon; 2: optic tectum; 3: cerebellum and 4: hypothalamus.


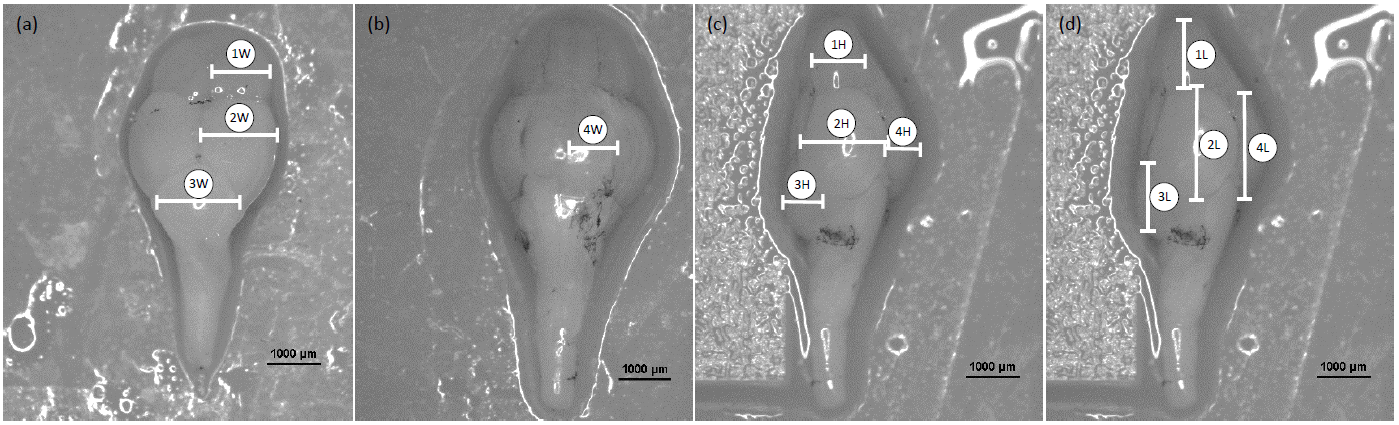


**Figure S4.** Correlation plot of whole brain and region volumes. Color intensity and the size of the circle are according to the value of the correlation coefficients. On the right, the legend shows the Spearman correlation coefficients and the corresponding colors.


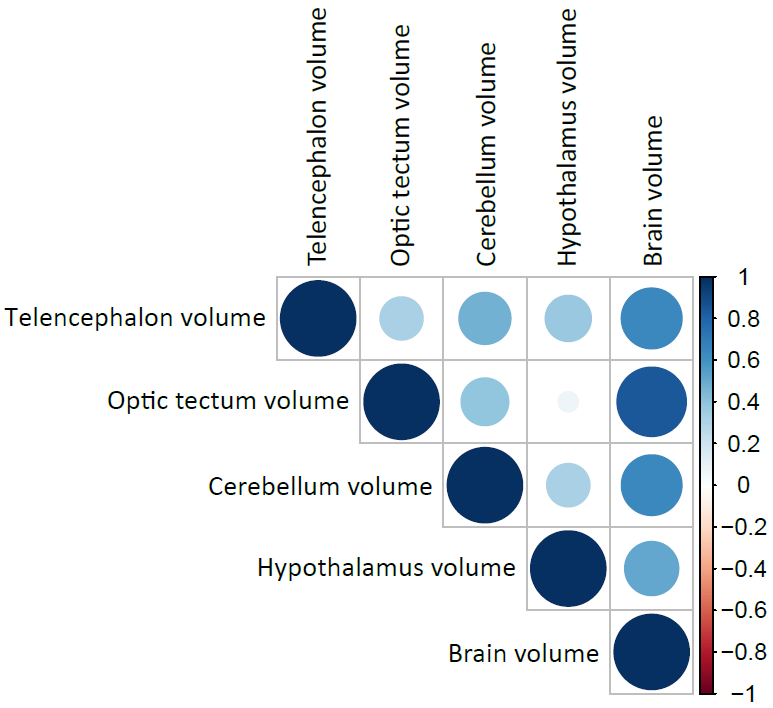


**Table S1.** Summary of the LMM analyses of cognitive ability traits, initial detour-reaching (i.e. initial score) and learning rate in the detour-reaching task, before and after the environmental complexity treatment. Significant p-values are highlighted in bold.

|  | Initial detour-reaching score (0.25 + log-transformed) before the treatment | | | | |  | Initial detour-reaching score (0.25 + log-transformed) after the treatment | | | | |  |
| --- | --- | --- | --- | --- | --- | --- | --- | --- | --- | --- | --- | --- |
|  | *β* | 95% CIs | df | *F* | *p* |  | *β* | 95% CIs | df | *F* | *p* |  |
| Intercept | 0.65 | 0.42, 0.87 |  |  |  |  | 0.76 | 0.56, 0.95 |  |  |  |  |
| Maternal age (young) | -0.09 | -0.53, 0.35 | 1, 22.12 | 0.13 | 0.716 |  | -0.10 | -0.28, 0.08 | 1, 17.21 | 1.69 | 0.211 |  |
| Treatment (control) | 0.05 | -0.20, 0.29 | 2, 109.50 | 2.31 | 0.104 |  | -0.11 | -0.30, 0.08 | 2, 35.89 | 2.52 | 0.094 |  |
| Treatment (simple) | 0.26 | -0.22, 0.34 |  |  |  |  | 0.09 | -0.10, 0.28 |  |  |  |  |
| Sex (male) | 0.11 | -0.04, 0.26 | 1, 108.09 | 3.04 | 0.084 |  | -0.08 | -0.24, 0.08 | 1, 97.86 | 0.72 | 0.398 |  |

|  | Learning rate before the treatment | | | | |  | Learning rate after the treatment | | | | |  |
| --- | --- | --- | --- | --- | --- | --- | --- | --- | --- | --- | --- | --- |
|  | *β* | 95% CIs | df | *F* | *p* |  | *β* | 95% CIs | df | *F* | *p* |  |
| Intercept | 0.79 | 0.65, 0.93 |  |  |  |  | 0.72 | 0.54, 0.91 |  |  |  |  |
| Maternal age (young) | -0.04 | -0.17, 0.09 | 1, 20.78 | 0.19 | 0.671 |  | -0.02 | -0.17, 0.12 | 1, 40.02 | 0.30 | 0.585 |  |
| Treatment (control) | 0.04 | -0.10, 0.17 | 2, 38.12 | 0.64 | 0.530 |  | -0.08 | -0.30, 0.15 | 2, 38.93 | 5.06 | 0.848 |  |
| Treatment (simple) | 0.08 | -0.06, 0.21 |  |  |  |  | 0.11 | -0.11, 0.33 |  |  |  |  |
| Test day (second) | -0.09 | -0.18, 0.00 | 2, 247.70 | 10.10 | <**0.001** |  | -0.19 | -0.34, -0.03 | 2, 197.71 | 5.06 | **0.007** |  |
| Test day (third) | -0.21 | -0.30, -0.12 |  |  |  |  | -0.19 | -0.35, -0.03 |  |  |  |  |
| Sex (male) | 9.75e-3 | -0.09, 0.11 | 1, 115.18 | 0.08 | 0.782 |  | -0.03 | -0.14, 0.08 | 1, 97.26 | 1.40 | 0.239 |  |
| Treatment (control) × Test day (second) | *-* | - | - | - | - |  | 0.22 | 0.00, 0.43 | 4, 197.71 | 2.43 | **0.048** |  |
| Treatment (control) × Test day (third) | - | - | - | - | - |  | 0.21 | -0.01, 0.43 | - | - | - |  |
| Treatment (simple) × Test day (second) | - | - | - | - | - |  | -0.05 | -0.26, 0.16 | - | - | - |  |
| Treatment (simple) × Test day (third) | - | - | - | - | - |  | -0.06 | -0.28, 0.15 | - | - | - |  |

**Table S2.** Summary of the GLMMs with a binomial error distribution and logit link function of analyses of group behaviors, physical activity (i.e. the maximum number of fish swimming), exploration (i.e. the maximum number of fish exploring), and aggressive interactions (i.e. the proportion of aggressive interactions). Significant p-values are highlighted in bold.

|  | Maximum number of fish swimming | | | | |  | Proportion of aggressive interactions | | | | |  |
| --- | --- | --- | --- | --- | --- | --- | --- | --- | --- | --- | --- | --- |
|  | *β* | 95% CIs | χ²₁ | *p* | *p*-adjusted |  | *β* | 95% CIs | χ²₁ | *p* | *p*-adjusted |  |
| Intercept | -0.33 | -0.62, -0.03 |  |  |  |  | -2.87 | -3.25, -2.50 |  |  |  |  |
| Maternal age (young) | -0.08 | -0.29, 0.14 | 0.48 | 0.487 | 0.598 |  | -0.02 | -0.30, 0.26 | 0.02 | 0.887 | 0.950 |  |
| Treatment (control) | 0.04 | -0.22, 0.31 | 1.39 | 0.498 | 0.598 |  | 0.57 | 0.23, 0.91 | 14.11 | **<0.001** | **0.003** |  |
| Treatment (simple) | -0.11 | -0.38, 0.16 | - | - |  |  | 5.14e-3 | -0.34, 0.35 | - | - |  |  |
| Sampling day (second) | -0.26 | -0.48, -0.04 | 5.57 | **0.018** | 0.054 |  | -0.23 | -0.44, -0.03 | 4.85 | **0.027** | 0.063 |  |
| Treatment week (second) | -0.03 | -0.32, 0.26 | 8.63 | **0.035** | 0.063 |  | 0.47 | 0.18, 0.77 | 25.96 | <**0.001** | <**0.001** |  |
| Treatment week (third) | -0.37 | -0.68, -0.06 | - | **-** | - |  | 0.76 | 0.47, 1.06 | - | - | - |  |
| Treatment week (fourth) | -0.31 | -0.62, 0.00 | - | **-** | - |  | 0.39 | 0.09, 0.69 | - | - | - |  |

|  | Maximum number of fish exploring | | | | |  |
| --- | --- | --- | --- | --- | --- | --- |
|  | *β* | 95% CIs | χ²₁ | *p* | *p*-adjusted |  |
| Intercept | -0.86 | -1.25, -0.47 |  |  |  |  |
| Maternal age (young) | -0.24 | -0.53, 0.05 | 2.62 | 0.106 | 0.166 |  |
| Treatment (simple) | -9.23e-3 | -0.30, 0.28 | 0.04e-1 | 0.950 | 0.950 |  |
| Sampling day (second) | -0.61 | -0.91, -0.32 | 16.75 | **<0.001** | <**0.001** |  |
| Treatment week (second) | 0.43 | 0.00, 0.85 | 8.48 | **0.037** | 0.074 |  |
| Treatment week (third) | 0.41 | -0.02, 0.84 | - | **-** | - |  |
| Treatment week (fourth) | 0.62 | 0.20, 1.05 | - | **-** | - |  |

**Table S3.** Summary of the LMMs of growth (i.e. body size and mass). Significant p-values are highlighted in bold.

|  | Growth (body size) | | | | |  | Growth (body mass) | | | | |  |
| --- | --- | --- | --- | --- | --- | --- | --- | --- | --- | --- | --- | --- |
|  | *β* | 95% CIs | df | *F* | *p* |  | *β* | 95% CIs | df | *F* | *p* |  |
| Intercept | -0.54 | -0.96, -0.12 |  |  |  |  | -0.26 | -0.67, 0.15 |  |  |  |  |
| Maternal age (young) | 0.14 | -0.29, 0.58 | 1, 18.86 | 0.41 | 0.528 |  | 0.06 | -0.37, 0.48 | 1, 19.81 | 0.35 | 0.558 |  |
| Treatment (control) | 0.33 | -0.03, 0.68 | 2, 40.87 | 1.67 | 0.202 |  | 0.20 | -0.16, 0.57 | 2, 115.74 | 0.63 | 0.532 |  |
| Treatment (simple) | 0.12 | -0.23, 0.47 | - | - | - |  | 0.05 | -0.31, 0.41 |  |  |  |  |
| Time (after treatment) | 0.83 | 0.72, 0.94 | 1, 106.95 | 211.44 | **<0.001** |  | 0.64 | 0.41, 0.88 | 1, 111.89 | 30.69 | **<0.001** |  |
| Sex (male) | -0.21 | -0.50, 0.08 | 1, 109.42 | 2.02 | 0.158 |  | -0.18 | -0.49, 0.12 | 1, 115.06 | 1.44 | 0.231 |  |
| Maternal age (young) × Time (after treatment) | - | - | - | - | - |  | -0.36 | -0.69, -0.03 | 1, 111.81 | 4.53 | **0.035** |  |

**Table S4.** Summary of the LMM analyses of survival. Significant p-values are highlighted in bold.

|  | Survival until the end of the study | | | |  | Survival until the end of the exposition to Apparatus 3 (i.e. 21 days) | | | |  |
| --- | --- | --- | --- | --- | --- | --- | --- | --- | --- | --- |
|  | *β* | 95% CIs | χ²₁ | *p* |  | *β* | 95% CIs | χ²₁ | *p* |  |
| Maternal age (young) | 0.10 | -0.74, 0.95 | 0.06 | 0.808 |  | -1.03 | -2.45, 0.39 | 2.02 | 0.155 |  |
| Treatment (control) | -0.39 | -1.37, 0.59 | 1.72 | 0.424 |  | -2.30 | -4.44, -0.16 | 6.39 | 0.040 |  |
| Treatment (simple) | -0.69 | -1.75, 0.36 |  |  |  | -1.40 | -2.98, 0.18 |  |  |  |
| Sex (male) | -0.52 | -1.41, 0.38 | 1.26 | 0.261 |  | -0.75 | -2.14, 0.64 | 1.112 | 0.292 |  |

**Table S5.** Summary of the LMM analyses of volumes of the whole brain and different brain regions. Significant p-values are highlighted in bold.

|  | Whole brain volume | | | | | |  | Optic tectum volume | | | | | |  |
| --- | --- | --- | --- | --- | --- | --- | --- | --- | --- | --- | --- | --- | --- | --- |
|  | *β* | 95% CIs | df | *F* | *p* | *p*-adjusted |  | *β* | 95% CIs | df | *F* | *p* | *p*-adjusted |  |
| Intercept | 0.27 | -0.22, 0.75 |  |  |  |  |  | 0.12 | -0.34, 0.57 |  |  |  |  |  |
| Maternal age | 0.05 | -0.44, 0.54 | 1, 19.65 | 0.04e-2 | 0.984 | 0.984 |  | -0.09 | -0.48, 0.30 | 1, 81.05 | 0.21 | 0.651 | 0.816 |  |
| Treatment (control) | -0.53 | -1.00, -0.07 | 2, 31.41 | 3.67 | **0.037** | 0.102 |  | -0.16 | -0.65, 0.32 | 2, 91.63 | 1.18 | 0.311 | 0.489 |  |
| Treatment (simple) | -0.32 | -0.75, 0.11 | - | - | - |  |  | -0.36 | -0.83, 0.11 | - | - | - |  |  |
| Sex | 0.08 | -0.29, 0.45 | 1, 77.68 | 2.66 | 0.107 | 0.214 |  | 0.25 | -0.14, 0.65 | 1, 91.55 | 1.63 | 0.204 | 0.367 |  |
| Body size | 0.48 | 0.29, 0.66 | 1, 73.67 | 33.39 | **<0.001** | **<0.001** |  | 0.37 | 0.18, 0.56 | 1, 92.00 | 14.42 | **<0.00**1 | **<0.001** |  |

|  |  | Hypothalamus volume | | | | | |  |
| --- | --- | --- | --- | --- | --- | --- | --- | --- |
|  |  | *β* | 95% CIs | df | *F* | *p* | *p*-adjusted |  |
| Intercept |  | 0.27 | -0.22, 0.75 |  |  |  |  |  |
| Maternal age |  | 0.05 | -0.44, 0.54 | 1, 19.37 | 0.04 | 0.833 | 0.916 |  |
| Treatment (control) |  | -0.53 | -1.00, -0.07 | 2, 81.93 | 2.66 | 0.076 | 0.167 |  |
| Treatment (simple) |  | -0.32 | -0.75, 0.11 | - | - | - |  |  |
| Sex |  | 0.08 | -0.29, 0.45 | 1, 82.00 | 0.18 | 0.668 | 0.816 |  |
| Body size |  | 0.48 | 0.29, 0.66 | 1, 83.92 | 24.99 | **<0.001** | **<0.001** |  |

|  | Telencephalon volume | | | | |  |  | Cerebellum volume | | | | | |  |
| --- | --- | --- | --- | --- | --- | --- | --- | --- | --- | --- | --- | --- | --- | --- |
|  | *β* | 95% CIs | df | *F* | *p* | *p*-adjusted |  | *β* | 95% CIs | df | *F* | *p* | *p*-adjusted |  |
| Intercept | 0.57 | 0.04, 1.10 |  |  |  |  |  | 0.55 | 0.00, 1.11 |  |  |  |  |  |
| Maternal age | -0.58 | -1.27, 0.11 | 1, 25.40 | 0.22 | 0.643 | 0.816 |  | -0.72 | -1.41, -0.02 | 1, 34.71 | 0.08 | 0.777 | 0.899 |  |
| Treatment (control) | -1.30 | -1.96, -0.64 | 2, 33.63 | 3.11 | 0.057 | 0.139 |  | -0.83 | -1.51, -0.15 | 2, 34.50 | 1.60 | 0.217 | 0.367 |  |
| Treatment (simple) | -0.66 | -1.29, -0.04 |  |  |  |  |  | -0.37 | -1.51, -0.18 | - | - | - |  |  |
| Sex | 0.17 | -0.18, 0.52 | 1, 86.33 | 0.89 | 0.349 | 0.512 |  | -0.02 | -0.43, 0.39 | 1, 80.79 | 0.05e-1 | 0.939 | 0.984 |  |
| Body size | 0.47 | 0.29, 0.64 | 1, 84.65 | 27.21 | **<0.001** | **<0.001** |  | 0.42 | 0.23, 0.62 | 1, 76.38 | 18.42 | **<0.001** | **<0.001** |  |
| Maternal age (young) × Treatment (control) | 1.50 | 0.57 2.42 | 2, 34.14 | 5.25 | **0.010** | **0.037** |  | 1.45 | 0.46, 2.43 | 2, 37.07 | 4.39 | **0.019** | 0.059 |  |
| Maternal age (young) × Treatment (simple) | 0.56 | -0.31, 1.43 | - | - | - | - |  | 0.88 | -0.07, 1.83 | - | - | - | - |  |
